# A bone optimization rheumatology clinic increases anabolic bone agent use and reduces mechanical complications in adult spinal deformity surgery

**DOI:** 10.1007/s43390-026-01307-z

**Published:** 2026-02-14

**Authors:** Harsh Jain, Advith Sarikonda, Ranbir Ahluwalia, Omar Zakieh, Austin Montgomery, Walter Navid, Philip Raj, Clayton R. Baker, Hani Chanbour, Iyan Younus, Tyler Zeoli, Soren Jonzzon, Autumn Zuckerman, S. Bobo Tanner, Julian G. Lugo-Pico, Amir M. Abtahi, Byron F. Stephens, Scott L. Zuckerman

**Affiliations:** 1 Department of Neurological Surgery, Vanderbilt University Medical Center, Medical Center North T-4224, Nashville, TN 37212 USA; 2 Department of Orthopedic Surgery, Vanderbilt University Medical Center, Nashville, TN USA; 3 School of Medicine, Vanderbilt University, Nashville, TN USA; 4 Vanderbilt Specialty Pharmacy, Vanderbilt University Medical Center, Nashville, TN USA; 5 Department of Rheumatology, Vanderbilt University Medical Center, Nashville, TN USA

**Keywords:** Scoliosis, Rheumatologist, Osteoporosis, Complications

## Abstract

**Purpose:**

Though the use of anabolic bone agents has proven effective in adult spinal deformity (ASD) surgery, prescription and approval of these medications remains a challenge. In osteopenic/osteoporotic patients undergoing ASD surgery, we sought to determine the impact of a bone optimization clinic on: (1) prescription patterns of anabolic agents, (2) mechanical complications, and (3) reoperation.

**Methods:**

A retrospective cohort study (2009–23) was performed for osteopenic/osteoporotic patients undergoing ASD surgery with ≥ 2-year follow-up. The study period was binarized into before/after a bone optimization clinic was established (2009–19 vs. 2020–23). The primary outcomes were: (1) use of anabolic agents prior to surgery (Teriparatide, Abaloparatide, and Romosozumab-aqqg), (2) mechanical complications, and (3) reoperation. Multivariable regression controlling for age, sex, body mass index, and operative time was performed.

**Results:**

Of 126 patients (mean age 68 ± 10yrs; 86% female) undergoing ASD surgery with osteopenia (80%) or osteoporosis (20%), 91 (72%) were before the bone optimization clinic and 35 (28%) were after. Similar rates of osteoporosis in both groups were seen (pre-21% vs. post-17%, *p* = 0.638). After the bone optimization clinic, more patients received preoperative anabolic therapy (54% vs. 23%, *p* < 0.001) and for a longer duration (98 ± 156 vs. 40 ± 109 days, *p* = 0.027). Overall mechanical complications decreased significantly (49% vs 81%, *p* < 0.001) as did reoperation for mechanical complications (6% vs. 44%, *p* < 0.001). Multivariable regression showed that a bone optimization clinic independently improved the use of preoperative anabolic agents (OR = 5.3, 95%CI:2.1–13.4, *p* < 0.001) and reduced the risk of mechanical complications (OR = 0.2 95%CI:0.1–0.5, *p* < 0.001) and reoperation for mechanical complications (OR = 0.1, 95%CI:0.1–0.4, *p* < 0.001).

**Conclusion:**

In osteopenic/osteoporotic patients undergoing ASD surgery, a bone optimization clinic was independently associated with increased prescription rates and duration of anabolic bone agents, reduced mechanical complications, and reduced reoperation for mechanical complications.

## Introduction

Successful outcomes in adult spinal deformity (ASD) surgery depend not only on achieving radiographic alignment but also on robust fixation and durable fusion [1]. In patients with compromised bone quality, such as those with osteopenia/osteoporosis, the structural integrity of the spinal construct is at risk, leading to higher rates of mechanical complications like hardware loosening, proximal junctional kyphosis/failure (PJK/F), and pseudarthrosis [2, 3]. As such, preoperative bone quality plays a crucial role in determining the success and durability of surgical reconstruction in the long term [4].

Thorough preoperative bone health screening and optimization are essential to prevent complications related to osteoporosis in ASD surgery, and initiating osteoanabolic therapy in selected patients can improve fusion and reduce mechanical complications [4–6]. In a study of patients undergoing ASD surgery, Mohanty et al.,[3] found that osteoporotic patients taking teriparatide experienced lower rates of reoperation and symptomatic pseudarthrosis compared to osteopenic patients [3]. Similarly, Pan et al.[7] performed a meta-analysis assessing outcomes for spinal fusion patients taking teriparatide, and found teriparatide was associated with higher rates of fusion and lower rates of screw loosening compared to control [7]. Osteoporotic patients have also been shown to have a lower axial pullout force of pedicle screws and higher risk of reoperation compared to patients with normal bone mineral density [3, 8–10]. Despite evidence supporting their effectiveness, the use of preoperative osteoanabolic agents remains inconsistent [2]. Barriers to initiating osteoanabolic therapy include complex approval processes, prescription challenges for surgeons who may be unfamiliar with the medications, and lack of access to metabolic health specialists such as rheumatologists [11, 12].

To streamline optimization of bone health in patients undergoing ASD surgery, our institution implemented a bone health optimization clinic with a dedicated rheumatologist in 2020, which optimized the bone health of patients with osteopenia/osteoporosis preoperatively. Therefore, in osteopenic/osteoporotic patients undergoing ASD surgery, we sought to determine the impact of a bone optimization clinic on: (1) prescription patterns of anabolic agents, (2) mechanical complications, and (3) reoperation.

## Methods

### Study design

A single-institution, retrospective cohort study was conducted of osteopenic/osteoporotic patients undergoing ASD surgery from 2009 to 2023. The patients in the registry were contributed by 5 fellowship-trained neurosurgical and orthopedic spine surgeons. Institutional Review Board approval was obtained, and patient consent was waived due to the study’s retrospective nature.

### Study population

**Fig. 1:**
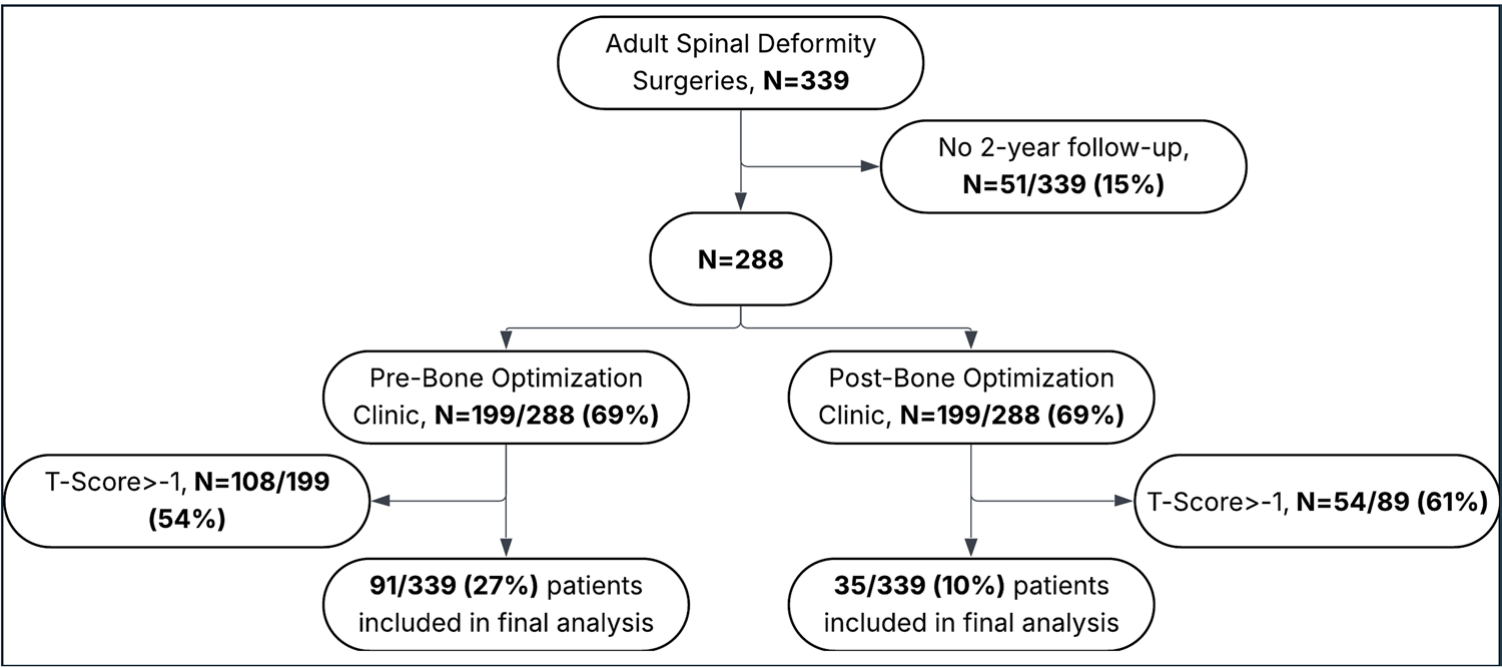
Flow-chart showing inclusion criteria for the study

Patients included in the study were osteopenic/osteoporotic adults undergoing elective surgery for ASD with ≥ 5 levels fused and ≥ 2-year follow-up. Patients were included if they fulfilled the following criteria: Cobb angle > 30°, coronal vertical axis (CVA) > 3 cm, sagittal vertical axis (SVA) > 5 cm, pelvic tilt (PT) of > 25°, thoracic kyphosis > 60°, or pelvic incidence (PI)–lumbar lordosis (LL) mismatch of > 10°. Osteopenia was defined as a T-score ≤ − 1.0, while a T-score ≤ -2.5 was considered osteoporotic [13, 14]. A flow-chart for inclusion of patients in the study is seen in Fig. 1.

At our institution, any patient undergoing a multi-level fusion gets a DEXA scan, and this scan has to be within 1-year of the intended surgery. Only patients with a documented T-score ≤ –1.0 (confirming osteopenia or osteoporosis) were eligible for referral to the bone optimization clinic and inclusion in this study.

### Exposure variables

The primary exposure variable was the time period of the surgery, binarized into before/after a bone optimization clinic was established (2009–19 vs. 2020–23). Following the establishment of the bone optimization clinic in 2020, all osteopenic and osteoporotic patients, as identified on preoperative DEXA scanning, were referred for rheumatology evaluation at the dedicated bone optimization clinic, without prioritization based on surgical risk or deformity severity. Additional patient demographic data was collected, including factors such as age, sex, body mass index (BMI), estimated blood volume and comorbidities. Perioperative variables included total number of instrumented levels, use of a 3-column osteotomy, operative time, estimated blood loss, hospital length of stay, and discharge home. The estimated blood volume was calculated using Nadler’s formula [15].

### Outcome variables

The primary outcomes were: (1) use and duration of anabolic agents prior to surgery (Teriparatide, Abaloparatide, and Romosozumab-aqqg), (2) mechanical complications, and (3) reoperation. Mechanical complications were defined in line with the prior literature [16, 17]. PJK occurred if there was a ≥ 10° increase in kyphosis between the upper instrumented vertebrae (UIV) and UIV + 2, while PJF occurred when PJK presented along with one or more of the following features: posterior osseo-ligamentous disruption, fracture of the vertebral body of the UIV or UIV + 1, or pullout of UIV instrumentation. [16, 18] Distal junctional kyphosis (DJK) was defined as a ≥ 10° increase in kyphosis between the lowest instrumented vertebra (LIV) and LIV-1 on postoperative radiographs. Implant failure included screw pullout, loosening, breakage, or dislodgement. Rod fracture was defined as the breakage of a single or double rod.

### Statistical analysis

Descriptive statistics were utilized to describe the study population. Shapiro–Wilk test was used to assess the normality of continuous variables. Continuous variables were described with mean ± SD, while categorical variables were described with frequencies and percentages. Independent t-tests were used to assess normally distributed data with equal variances, while Wilcoxon signed-rank test or the Mann–Whitney-U test were used to assess nonparametric data. For categorical data, the Chi-Square or Fisher’s exact test was applied for small samples. Univariate and multivariable regression, controlled for age, sex, BMI, and operative time, were performed. All statistical tests were conducted using IBSM SPSS Statistics version 29 (IVM Corp), and statistical significance was achieved if *p* < 0.05.

## Results

### Population characteristics

A total of 126 osteopenic/osteoporotic patients undergoing adult spinal deformity (ASD) surgery were included. The mean age was 68.2 ± 10.4 years, and the majority were female (N = 108, 85.7%). Of the total, 91 patients (72.2%) underwent surgery prior to the implementation of the bone optimization clinic (2009–2019), while 35 patients (27.8%) were treated after the clinic was established (2020–2023).

Demographic and baseline characteristics were similar between the pre- and post-clinic groups, with no significant differences in age (69.5 ± 10.7 vs. 65.0 ± 8.7 years, *p* = 0.062), sex (male: 12.1% vs. 20.0%, *p* = 0.256; 11 vs. 7 patients), BMI (27.6 ± 6.2 vs. 28.9 ± 5.3, *p* = 0.275), comorbidity burden (*p* = 0.640), or rates of osteopenia (79.1% vs. 82.9%, *p* = 0.638; 72 vs. 29 patients) and osteoporosis (20.9% vs. 17.1%, *p* = 0.638; 19 vs. 6 patients). The demographic characteristics are summarized in Table 1.

**Table 1:** Demographic characteristics and perioperative variables

Character	After bone optimization clinic, N = 35	Before bone optimization clinic, N = 91	*P* value
Age (years), mean ± SD	65.0 ± 8.7	69.5 ± 10.7	0.062
Sex, male, n (%)	7 (20.0%)	11 (12.1%)	0.256
BMI, mean ± SD	28.9 ± 5.3	27.6 ± 6.2	0.275
Comorbidities, n (%) 0 1 2 +	6 (17.1%) 12 (34.3%) 17 (48.6%)	20 (22.0%) 35 (38.5%) 36 (39.6%)	0.640
Diabetes, n (%)	14 (15.4%)	9 (25.7%)	0.179
Chronic obstructive lung disease, n (%)	31 (34.1%)	8 (22.9%)	0.223
Heart failure, n (%)	11 (12.1%)	10 (28.6%)	**0.026**
Hypertension, n (%)	28 (80.0%)	62 (68.1%)	0.187
Osteoporosis, n (%)	6 (17.1%)	19 (20.9%)	0.638
Osteopenia, n (%)	29 (82.9%)	72 (79.1%)	0.638
Preoperative anabolic therapy, n (%)	19 (54.3%)	21 (23.3%)	** < 0.001**
Duration of anabolic therapy (days), mean ± SD	98.1 ± 156.0	40.0 ± 108.5	**0.027**
Romosozumab-aqqg, n (%)	4 (11.4%)	0 (0.0%)	**0.001**
Teriparatide, n (%)	13 (37.1%)	37 (40.7%)	0.718
Abaloparatide, n (%)	10 (28.6%)	2 (2.2%)	** < 0.001**
3-column osteotomy, n (%)	7 (20.0%)	19 (20.9%)	0.913
Procedure Posterior only Anterior–Posterior	18 (51.4%) 17 (48.6%)	79 (86.8%) 12 (13.2%)	** < 0.001**
Total instrumented levels, mean ± SD	11.7 ± 4.2	10.5 ± 2.8	0.062
Estimated blood volume (mL), mean ± SD	4176.6 ± 847.0	4103.4 ± 831.3	0.661
Estimated blood loss (mL), mean ± SD	730.4 ± 527.0	1610.3 ± 1390.3	** < 0.001**
Blood loss/bloss volume (%), mean ± SD	17.2 ± 10.3	39.7 ± 34.3	** < 0.001**
Operative time (min), mean ± SD	448.3 ± 159.0	421.8 ± 143.9	0.370
Length of stay (days), mean ± SD	9.3 ± 11.6	8.1 ± 13.6	0.655
Discharge home, n (%)	18 (54.5%)	40 (45.5%)	0.373
Postoperative anabolic therapy, n (%)	18 (51.4%)	33 (36.7%)	0.132
Postoperative duration of anabolic therapy (days), mean ± SD	230.0 ± 307.8	128.7 ± 217.8	**0.041**

### Perioperative characteristics

There were no significant differences in the rates of use of 3-column osteotomy (20.0% vs. 20.9%, *p* = 0.913; 7 vs. 19 patients) or total instrumented levels (11.7 ± 4.2 vs. 10.5 ± 2.8, *p* = 0.062). The patients treated after the implementation of the clinic had significantly higher rate of anterior–posterior procedures (48.6% vs. 13.2%, *p* < 0.001; 17 vs. 12 patients), with no significant differences in operative time (448.3 ± 159.0 vs. 421.8 ± 143.9 min, *p* = 0.370), hospital length of stay (9.3 ± 11.6 vs. 8.1 ± 13.6 days, *p* = 0.655), or discharge home (54.5% vs. 45.5%, *p* = 0.373; 18 vs. 40 patients) (Table 1).

### Preoperative anabolic therapy

Following the establishment of the bone optimization clinic, the proportion of patients receiving preoperative anabolic therapy significantly increased (54.3% vs. 23.3%, *p* < 0.001; 19 vs. 21 patients). The duration of therapy was also longer in the post-clinic group (98.1 ± 156.0 vs. 40.0 ± 108.5 days, *p* = 0.027) **(**Table 1**)**.

Postoperatively, although the rate of anabolic therapy was similar (51.4% vs. 36.7%, *p* = 0.132; 18 vs. 33 patients), the post-clinic cohort continued therapy for a longer duration (230.0 ± 307.8 vs. 128.7 ± 2178 days, *p* = 0.041).

### Mechanical complications and reoperation

Mechanical complications were significantly lower in the post-clinic group (48.6% vs. 81.3%, *p* < 0.001; 17 vs. 74 patients), as were reoperations (22.9% vs. 45.1%, *p* = 0.022; 8 vs. 41 patients) and reoperations specifically for mechanical complications (5.7% vs. 44.0%, *p* < 0.001; 2 vs. 40 patients). Rates of radiographic PJK (36.4% vs. 62.2%, *p* = 0.011; 12 vs. 56 patients), pseudarthrosis (5.7% vs. 41.8%, *p* < 0.001; 2 vs. 38 patients), and spinopelvic complications (11.4% vs. 46.2%, *p* < 0.001; 4 vs. 42 patients) were also significantly lower in the post-clinic cohort. There were no significant differences in rod fracture (17.1% vs. 27.5%, *p* = 0.228; 6 vs. 25 patients), distal junctional kyphosis (DJK) (2.9% vs. 2.2%, *p* = 1.000; 1 vs. 2 patients), or implant failure (14.3% vs. 8.8%, *p* = 0.348; 5 vs. 8 patients) **(**Table 2**)**.

**Table 2:** Postoperative outcomes

Character	After bone optimization clinic, N = 35	Before bone optimization clinic, N = 91	*P* value
Reoperation, n (%)	8 (22.9%)	41 (45.1%)	**0.022**
Mechanical complication, n (%)	17 (48.6%)	74 (81.3%)	** < 0.001**
Mechanical complication requiring reoperation, n (%)	2 (5.7%)	40 (44.0%)	** < 0.001**
Radiographic PJK, n (%)	12 (36.4%)	56 (62.2%)	**0.011**
DJK, n (%)	1 (2.9%)	2 (2.2%)	1.000
Pseudarthrosis, n (%)	2 (5.7%)	38 (41.8%)	** < 0.001**
Rod fracture, n (%)	6 (17.1%)	25 (27.5%)	0.228
Implant failure, n (%)	5 (14.3%)	8 (8.8%)	0.348
Spinopelvic complications, n (%)	4 (11.4%)	42 (46.2%)	** < 0.001**

### Multivariable analysis

On multivariable logistic regression, controlling for age, sex, BMI and operative time, the implementation of a bone optimization clinic was independently associated with a significantly increased likelihood of receiving preoperative anabolic therapy (OR = 5.33, 95% CI: 2.13–13.38, *p* < 0.001), for a longer duration (β = 62.89, 95%CI:9.40–116.39, *p* = 0.022). It was also associated with a significantly lower risk of mechanical complications (OR = 0.21, 95% CI: 0.09–0.52, *p* < 0.001), overall reoperation (OR = 0.34, 95% CI: 0.14–0.86, *p* = 0.022), and reoperation specifically for mechanical complications (OR = 0.08, 95%CI: 0.02–0.35, *p* < 0.001). Specifically, the odds of radiographic PJK (OR = 0.35, 95%CI: 0.14–0.83, *p* = 0.018), pseudarthrosis (OR = 0.09, 95%CI: 002–0.39), and spinopelvic complications (OR = 0.15, 95%CI: 0.05–0.48, *p* < 0.001), reduced significantly **(**Table 3**)**.

**Table 3:** Multivariable regression controlling for age, sex, BMI and operative time

Outcome	Independent variable	OR/β (95%CI)	*P*-value
Preoperative anabolic therapy	Bone Optimization Clinic	5.33 (2.13–13.38)	** < 0.001**
Duration of preoperative anabolic therapy		62.89 (9.40–116.39)	**0.022**
Reoperation		0.34 (0.14–0.86)	**0.022**
Mechanical complications		0.21 (0.09–0.52)	** < 0.001**
Mechanical complication requiring reoperation		0.08 (0.02–0.35)	** < 0.001**
Radiographic PJK		0.35 (0.14–0.83)	**0.018**
Pseudarthrosis		0.09 (0.02–0.39)	**0.002**
Rod fracture		0.59 (0.21–1.65)	0.313
Implant failure		1.80 (0.52–6.21)	0.350
Spinopelvic complications		0.15 (0.05–0.48)	**0.001**

### Sub-analysis of patients operated in 2019 versus 2020

**Fig. 2:**
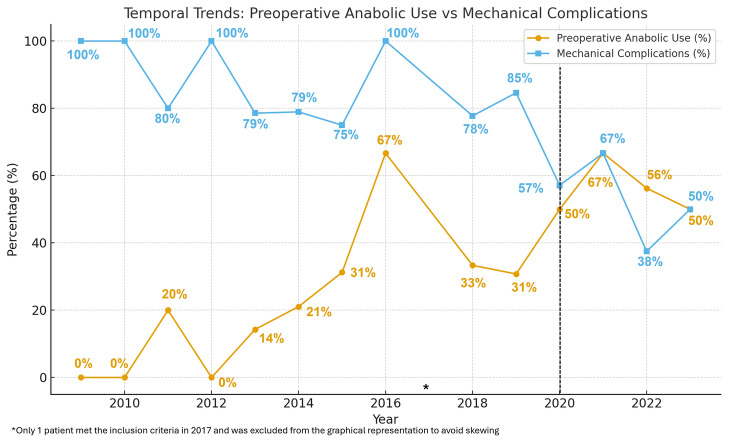
Temporal trends of preoperative anabolic therapy and postoperative mechanical complications

To mitigate the effect of improved surgical techniques during the study time period, we compared the patients operated immediately before (2019) to those immediately after (2020) the establishment of the bone optimization clinic. Although limited by a small sample size, several notable trends emerged. The patients who underwent surgery in 2020 had a higher rate of preoperative anabolic therapy (50.0% vs. 33.3%, *p* = 0.391; 7 vs. 4 patients), for a longer duration (84.7 ± 136.8 vs. 77.3 ± 228.6 days, *p* = 0.919), although it did not reach significance. However, postoperative outcomes improved markedly. The 2020 cohort demonstrated significantly lower rates of overall reoperation (7.1% vs. 46.2%, *p* = 0.033; 1 vs. 6 patients), reoperation for mechanical complications (0.0% vs. 46.2%, *p* = 0.006; 8 vs. 11 patients), and spinopelvic complications (14.2% vs. 53.8%, *p* = 0.046; 2 vs. 7 patients). Similar reductions were observed across other mechanical complication categories, though these trends did not reach statistical significance due to limited sample size and power (Table 4). The temporal trends of preoperative anabolic use and overall mechanical complications are shown in Fig. 2.

**Table 4:** Comparison of patients operated immediately before (2019) and after (2020) the establishment of the bone optimization clinic

Character	2019, N = 13	2020, N = 14	*P* value
Preoperative anabolic therapy, n (%)	4 (33.3%)	7 (50.0%)	0.391
Duration of preoperative anabolic therapy (days), mean ± SD	77.3 ± 228.6	84.7 ± 136.8	0.919
Reoperation, n (%)	6 (46.2%)	1 (7.1%)	**0.033**
Mechanical complication, n (%)	11 (84.6%)	8 (57.1%)	0.209
Mechanical complication requiring reoperation, n (%)	6 (46.2%)	0 (0.0%)	**0.006**
Radiographic PJK, n (%)	8 (66.7%)	6 (46.2%)	0.302
DJK, n (%)	0 (0.0%)	1 (7.1%)	1.000
Pseudarthrosis, n (%)	6 (46.2%)	0 (0.0%)	**0.006**
Rod fracture, n (%)	5 (38.5%)	4 (28.6%)	0.695
Implant failure, n (%)	3 (23.1%)	0 (0.0%)	0.098
Spinopelvic complications, n (%)	7 (53.8%)	2 (14.3%)	**0.046**

## Discussion

The current study evaluated the impact of a dedicated bone optimization clinic on prescription of anabolic agents and mechanical complications in osteopenic/osteoporotic patients undergoing ASD surgery. Following the establishment of a bone optimization clinic, the proportion of patients receiving preoperative anabolic therapy increased, and therapy duration was longer. Importantly, the post-clinic cohort experienced substantially lower rates of mechanical complications, reoperations, and complications such as pseudarthrosis, spinopelvic complications, and PJK. These findings highlight the clinical impact of a structured bone optimization program in improving surgical outcomes for osteopenic/osteoporotic patients undergoing ASD surgery.

The establishment of a dedicated bone optimization clinic nearly doubled both the prescription rates and preoperative duration of anabolic therapy, in osteopenic/osteoporotic patients undergoing ASD surgery. In line with our results, Riley et al.[19] evaluated osteoporotic patients and concluded that a bone health clinic had a significantly higher rate of prescription of anabolic bone agents compared to other physicians [19]. Although prior literature emphasizes the efficacy of osteoanabolic agents in enhancing fusion and reducing complications in spine surgery patients with poor bone quality [3, 4, 11], initiation of these therapies can be delayed by complex approval processes, prescribing challenges for surgeons unfamiliar with these medications, and limited access to metabolic specialists [11, 12]. Adhering to practice guidelines, the efficacy of anabolic agents is the highest when initiated at least 1 to 3 months before surgery [20–22]. A bone health optimization clinic with a dedicated rheumatologist facilitates direct collaboration with metabolic specialists, ensuring timely evaluation and individualized therapy initiation, while clinical pharmacists streamline insurance approvals and support patient adherence [23]. Importantly, the observed increase in anabolic use is largely attributable to systematic referral of eligible patients to this well-equipped clinic, likely for four reasons. First, we had a willing provider who wanted to collaborate and open up clinic spots, which improved access. Second, several lab values need to be obtained prior to safely starting anabolic therapy, and the rheumatology clinic had the expertise to do that. Third, these medications are expensive and hard to get approved by insurance. The rheumatology clinic, with the help of an experienced specialty pharmacist, was paramount in medication approval, specifically navigating prior authorizations, appeals, and insurance approval processes–key barriers previously limiting access to anabolic medications. Fourth, for patients that did not have appropriate insurance, the rheumatology clinic could give out free samples when appropriate. Due to the approval process and complex monitoring required of these powerful anabolic agents, spine surgeons alone cannot—and should not—be prescribing these medications. Notably, anabolic therapy was initiated preoperatively whenever feasible, with the goal of starting the treatment at least 12 weeks before surgery in accordance with institutional practice and the recommendation of the rheumatologist. The wide variability in duration reflects real-world factors such as differences in referral timing, insurance approval delays, and scheduling constraints, different medication mechanism, and perhaps most importantly, the lack of consensus within the spine community regarding preoperative duration of anabolic medication.

The observed reduction in mechanical complications following preoperative bone health optimization underscores the clinical value of integrating metabolic assessment and osteoanabolic therapy into surgical planning. Patients receiving targeted intervention showed lower rates of instrumentation failure, pseudoarthrosis, and PJK—complications closely linked to poor bone quality [4, 24]. Anabolic agents enhance bone formation and trabecular structure, promoting stronger fusion and improved construct stability, thereby reducing hardware-related failures, particularly in compromised bone [4, 24]. Given their heightened susceptibility to postoperative complications due to the compromised bone quality [25, 26], osteopenic/osteoporotic patients benefit most from preoperative bone optimization. Outside of spine surgery, various surgical subspecialties employ dedicated preoperative optimization clinics to improve patients’ readiness for surgery [27–29]. Barriers to widespread adoption of similar optimization clinics in spine surgery include a lack of standardized protocols, limited provider awareness, and logistical challenges [30]. By demonstrating the efficacy of a bone optimization clinic in increasing prescription of anabolic therapy and subsequently reducing mechanical complications, our study highlights the benefits of formal protocols for optimizing patients prior to spine surgery.

Our study has limitations which deserve mention. In addition to being a retrospective, single-institution study, our study binarizes the implementation of a rheumatology clinic into two epochs: 2009–19 versus 2020–23. While this design allowed us to evaluate changes following clinic initiation, it also limits broader generalizability, as the presence of a dedicated, multidisciplinary bone optimization program may not be feasible in many institutions, particularly those in practicing at smaller institutions or private practices. Importantly, it is plausible that the decrease in mechanical complications over this period may be due to improvements in surgical technique and/or a better overall awareness of the importance of preoperative bone optimization. Although we did compare patients undergoing surgery in 2019 versus 2020 to mitigate the effects of improved surgical techniques during the study time period, future studies should control for these biases through prospective, multicenter analyses that evaluate the impact of comprehensive prehabilitation efforts to reduce postoperative morbidity. Importantly, at our institution, the use of advanced intraoperative technologies that could substantially alter construct biomechanics has remained limited, with navigation used sparingly in ASD surgery and no cases performed with robotic assistance throughout the study period, as our institution does not own a robot. Additionally, the relatively small post-clinic cohort limits statistical power and generalizability; however, we believe these findings have potential to be clinically meaningful and should be interpreted as hypothesis-generating, warranting validation in larger multicenter studies.

## Conclusion

The implementation of a bone optimization clinic was independently associated with increased prescription rates and duration of anabolic bone agents for osteopenic/osteoporotic patients undergoing ASD surgery, which in turn was associated with reduced mechanical complications.

## Data Availability

The dataset generated and/or analyzed during the current study is not publicly available but may be obtained fromthe corresponding author on reasonable request.
